# Freeze-Dried Secretome (Lyosecretome) from Mesenchymal Stem/Stromal Cells Promotes the Osteoinductive and Osteoconductive Properties of Titanium Cages

**DOI:** 10.3390/ijms22168445

**Published:** 2021-08-06

**Authors:** Elia Bari, Fulvio Tartara, Fabio Cofano, Giuseppe di Perna, Diego Garbossa, Sara Perteghella, Marzio Sorlini, Delia Mandracchia, Lorella Giovannelli, Paolo Gaetani, Maria Luisa Torre, Lorena Segale

**Affiliations:** 1 Department of Drug Sciences, University of Pavia, Viale Taramelli 12, 27100 Pavia, Italy; elia.bari@unipv.it (E.B.); sara.perteghella@unipv.it (S.P.); 2 Fondazione IRCCS Istituto Neurologico Nazionale Mondino, Via Mondino 2, 27100 Pavia, Italy; tartarafulvio@gmail.com; 3 Neuroscience Department, “Rita Levi Montalcini”, Via Cherasco 15, 10126 Torino, Italy; fabio.cofano@gmail.com (F.C.); dr.giuseppediperna@gmail.com (G.d.P.); dgarbossa@gmail.com (D.G.); 4 Vertebral Surgery Unit, Humanitas Gradenigo, Corso Regina Margherita 8, 10153 Turin, Italy; 5 PharmaExceed S.r.l., Piazza Castello 19, 27100 Pavia, Italy; marzio.sorlini@supsi.ch (M.S.); neuro.gaetani@gmail.com (P.G.); 6 SUPSI—Department of Innovative Technologies, Lugano University Centre, Campus Est, Via la Santa 1, 6962 Viganello, Switzerland; 7 Department of Molecular and Translational Medicine, University of Brescia, Viale Europa 11, 25123 Brescia, Italy; delia.mandracchia@unibs.it; 8 Department of Pharmaceutical Sciences, University of Piemonte Orientale, Largo Donegani 2/3, 28100 Novara, Italy; lorella.giovannelli@uniupo.it (L.G.); lorena.segale@uniupo.it (L.S.); 9 U.O. Chirurgia Vertebrale, Istituto Clinico Città di Pavia, Gruppo San Donato, 27100 Pavia, Italy

**Keywords:** mesenchymal stem cells (MSCs), MSC-secretome, MSC-extracellular vesicles, bone regeneration

## Abstract

Titanium is one of the most frequently used materials in bone regeneration due to its good biocompatibility, excellent mechanical properties, and great osteogenic performance. However, osseointegration with host tissue is often not definite, which may cause implant failure at times. The present study investigates the capacity of the mesenchymal stem cell (MSC)-secretome, formulated as a ready-to-use and freeze-dried medicinal product (the Lyosecretome), to promote the osteoinductive and osteoconductive properties of titanium cages. In vitro tests were conducted using adipose tissue-derived MSCs seeded on titanium cages with or without Lyosecretome. After 14 days, in the presence of Lyosecretome, significant cell proliferation improvement was observed. Scanning electron microscopy revealed the cytocompatibility of titanium cages: the seeded MSCs showed a spread morphology and an initial formation of filopodia. After 7 days, in the presence of Lyosecretome, more frequent and complex cellular processes forming bridges across the porous surface of the scaffold were revealed. Also, after 14 and 28 days of culturing in osteogenic medium, the amount of mineralized matrix detected by alizarin red was significantly higher when Lyosecretome was used. Finally, improved osteogenesis with Lyosecretome was confirmed by confocal analysis after 28 and 56 days of treatment, and demonstrating the production by osteoblast-differentiated MSCs of osteocalcin, a specific bone matrix protein.

## 1. Introduction

Regeneration of bone defects often presents significant challenges. According to some reports, between 5% and 10% of all bone fractures result in delayed or failed healing [1,2]. This aspect is particularly evident for patients with decreased tissue regeneration capacity as a consequence of advanced age, disease, or extensive injury. In previous years, many research studies have extensively proved the efficacy of mesenchymal stem cells (MSCs), derived from various connective tissues, in enhancing bone regeneration through in vitro, in vivo, and clinical trials [3]. Early scientific investigations explained the effectiveness of MSCs through their ability to colonize the bone lesion and then differentiate, becoming bone-forming osteoblasts that replace the damaged resident osteoblasts [4]. Collectively, current research appears to instead argue that MSC paracrine activities play a more predominant role in bone tissue regeneration than differentiation [5]. It has been demonstrated that once MSCs are injected into a damaged bone, they show a relatively poor rate of cell engraftment, and the engrafted cells are short-lived and thus cannot differentiate into osteoblastic lineage [5,6]. Conversely, MSCs secrete cytokines, chemokines, and growth factors, both as free proteins and encapsulated into nano/microstructured extracellular vesicles (EVs), to orchestrate tissue repair. In detail, MSC-secretome can promote angiogenesis and tissue regeneration [7,8,9], inhibit fibrosis [10,11,12,13,14], and inflammation [15,16,17,18]. Furthermore, MSC-secretome can stimulate the tissue-resident MSCs to proliferate, leading to new and organized tissue formation and mineralizing, showing a higher expression of osteoblast markers and a high quantity of newly formed trabeculae [19,20]. Finally, MSC-secretome also has the advantage of being easily isolated from cell culture supernatants by scalable and GMP-compliant manufacturing procedures and formulated in a ready-to-use, freeze-dried pharmaceutical dosage form [21,22].

In bone tissue engineering, MSCs and MSC-secretome are often combined with scaffolds to improve bone reconstruction. In this regard, biomaterials that induce MSC-resident osteoblastic lineage differentiation without any exogenous chemical stimuli and capable of mimicking the physicochemical and mechanical properties of the bone extracellular matrix should be preferred. Titanium is one of the most commonly used biomaterials due to its good biocompatibility, mechanical properties, and osteogenic performance [23,24,25]. A recent work proved the superior osteoinductive and osteoconductive properties of scaffolds manufactured from titanium vs polyetheretherketone, which is another widely used biomaterial [26]. Usually, titanium is shaped as a mesh that is easily moldable and adaptable to the shape of the defect. While the shaping step is traditionally performed during surgery, new layer-by-layer deposition methods, such as the electron beam melting technique, shape the meshes on patients’ bone defects, with clear advantages over surgery time, patient discomfort, and morbidity. Despite titanium being one of the most frequently used materials in orthopedic implants, osseointegration with the host tissue is often not definite, which may cause implant failure at times [27].

In light of these data, the present study investigates the capacity of MSC-secretome formulated as a ready-to-use and freeze-dried medicinal product known as Lyosecretome to promote the osteoinductive and osteoconductive properties of titanium cages, and ultimately the osseointegration of scaffolds used in vivo. MSCs were seeded onto titanium cages and exposed, in the presence of the osteogenic medium, to Lyosecretome. The proliferation rate, production of calcified extracellular matrix, and the production of specific bone matrix proteins by osteoblast-differentiated MSCs, such as osteocalcin (OCN), were evaluated. To this end, several techniques, including confocal and scanning electron microscopy (SEM) and biochemical assays, were used.

## 2. Results and Discussion

Titanium, especially porous/trabecular implants, is the most frequently used material for treating bone defects [28], mainly for its osteoinductive and osteoconductive capacity [26,29]. This work investigated the ability of MSC-secretome in supporting cell adhesion, proliferation, and osteogenic differentiation of MSCs seeded on titanium cages, thus introducing next-generation tissue-engineered implants with enhanced biological performance. MSC-secretome was converted into a freeze-dried pharmaceutical dosage form (Lyosecretome) that can be easily adapted by the clinical community [10,15,21,22]. The Lyosecretome contained 22.00 ± 3.09 µg of proteins and 3.44 ± 0.07 µg of lipids per mg of powder. The EV mean diameter was 164.6 ± 8.5 nm, and the d_10_, d_50_, and d_90_ were 102.8 ± 4.5, 154.8 ± 6.1 and 298.7 ± 29.9 nm, respectively. In addition, Lyosecretome is free of bacteria (including mycoplasma), and its bacterial endotoxin level is in an acceptable range for the injectable dosage forms (lower than 3.4 Eu/mL). These results agree with our previous works [19,22], thus indicating the robustness of the preparation method.

**Figure 1:**
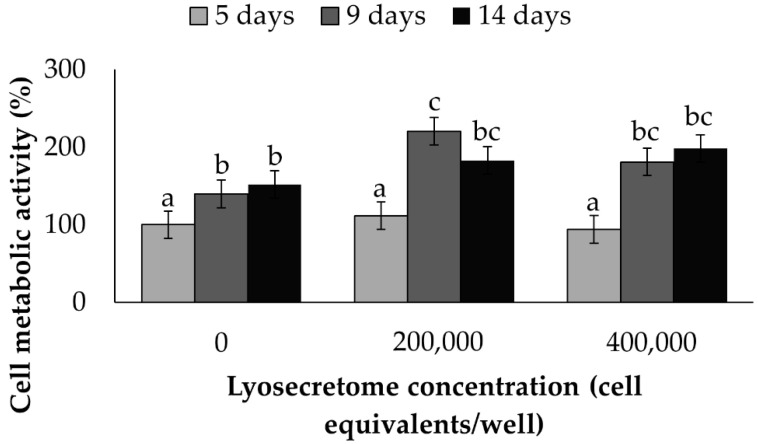
Cell metabolic activity (%) of MSCs grown on the scaffold cultured in serum-free medium and exposed to different doses of Lyosecretome over time. Untreated cells (0 Lyosecretome cell equivalents and no FBS) at the 5 day-time point were considered control. Multifactor ANOVA (time and concentration, mean values ± LSD, *n* = 3) was used. Both time and concentration were significant (*p* < 0.0001 and *p* = 0.0004, respectively). Different letters indicate a significant difference between the means (*p* < 0.05), while the same letters indicate no significant difference between the means (*p* > 0.05).

At first, the effect of increasing the dosage of Lyosecretome on the cell metabolic activity of MSCs cultured on the cages was investigated as a function of time (Figure 1). Both time and concentration were significant (*p* < 0.0001 and *p* = 0.0004, respectively). After 5 days of treatment, no differences were observed among the different Lyosecretome concentrations tested (*p* < 0.05), whereas after 9 and 14 days of treatment, 200,000 cell equivalents of Lyosecretome significantly increased the cell metabolic activity percentage (*p* < 0.05). There was no observable differences between the doses of 200,000 and 400,000 cell equivalents/per well (*p* > 0.05). These results confirm the cytocompatibility of Lyosecretome and its proliferative potential on MSCs, which was previously demonstrated on tenocytes and chondrocytes [30]. Overall, the ability of MSC-secretome to stimulate cell growth and proliferation may be linked to its content in mitogens and growth factors, such as insulin growth factor (IGF-1), vascular endothelial growth factor (VEGF), transforming growth factor β (TGF-β), platelet-derived growth factor (PDGF), fibroblast growth factor (FGF), and epidermal growth factor (EGF) [31,32]. Furthermore, in our previous work [21] using proteomic analysis, we demonstrated the presence of other proteins in Lyosecretome that can stimulate cell proliferation, such as fibronectin [33,34], complement C3 [35], and alpha-2 macroglobulin [36].

**Figure 2:**
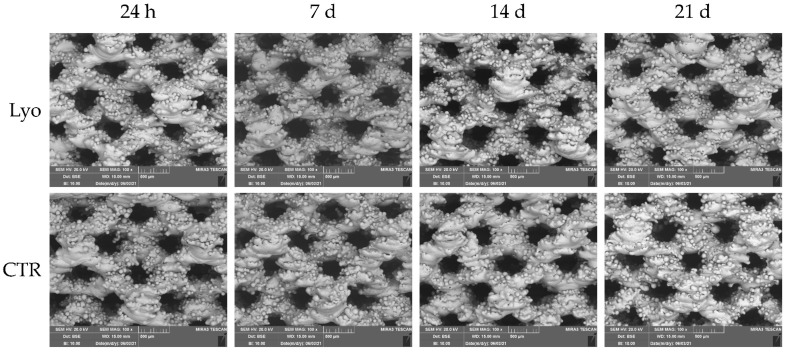
SEM morphological and structural characterizations of Ti cages seeded with MSCs and cultured in osteogenic medium with Lyosecretome or without (CTR). Images were acquired in backscattering mode to show the distribution of various elements that constitute the sample. The brighter regions reveal high-density materials (Ti) and the shaded areas indicate low-density materials (organic material). Magnification 100×. Scale bar: 500 µm.

MSCs are multipotent stem cells that can differentiate toward adipogenic, chondrogenic, and osteogenic cells when cultured in the appropriate culture media [37]. Thus, the effect of osteogenic culture medium supplemented with Lyosecretome (tested at 2 × 10^6^ cell equivalents per well) on the osteogenic differentiation of MSCs seeded on the cages was investigated as a function of time. Titanium cages showed a trabecular surface with a regular three-dimensional porous structure (Figure 2). Using the electron backscattered diffraction mode of SEM, the deposition of organic material onto the cages was monitored over time. After 7, 14, and 21 days, the brightest portions of scaffolds (which correspond to high-density materials, the Ti) were progressively shaded due to the deposition of organic material, which appears less bright (since it is less dense than Ti). This effect was particularly detectable after the treatment with Lyosecretome, especially after 7 days (Figure 2), and possibly masked by the fact that cells mainly grow into the pores of the scaffold [38].

**Figure 3:**
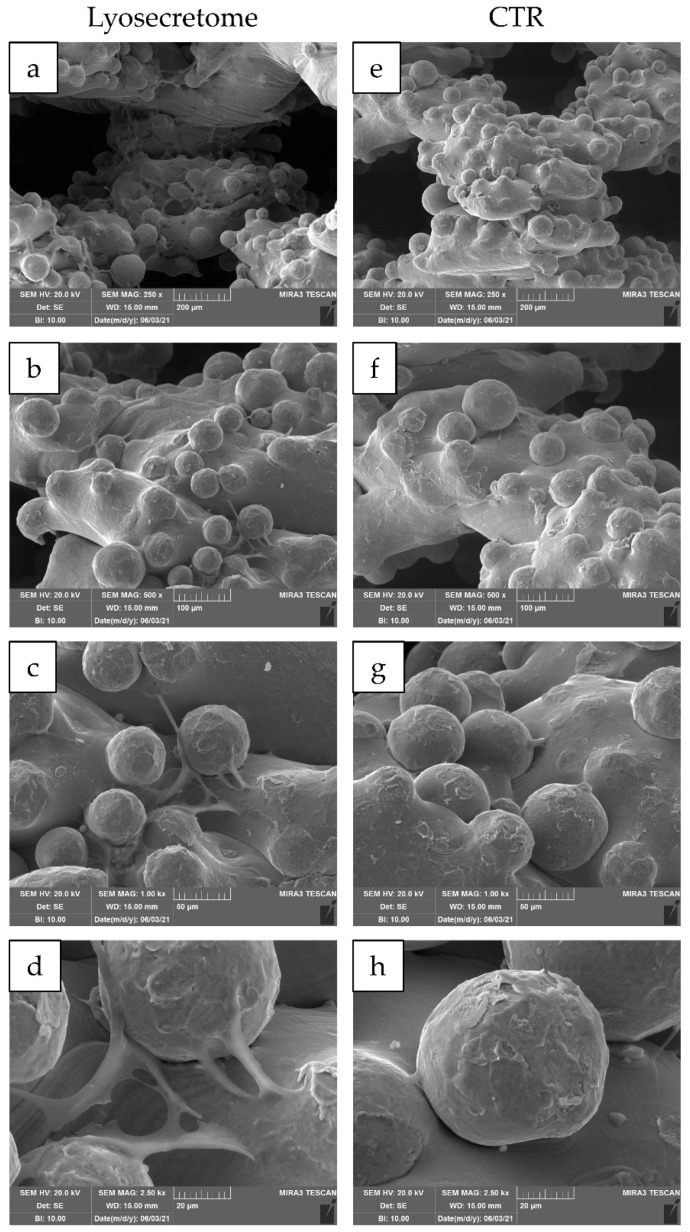
SEM morphological and structural characterizations of titanium cages seeded with MSCs and cultured in osteogenic medium with Lyosecretome or without (CTR) for 24 h. Magnifications: 250× (**a**,**e**), 500× (**b**,**f**), 1000× (**c**,**g**) and 2500× (**d**,**h**). Scale bars: 200 (**a**,**e**), 100 (**b**,**f**), 50 (**c**,**g**) and 20 (**d**,**h**) µm.

**Figure 4:**
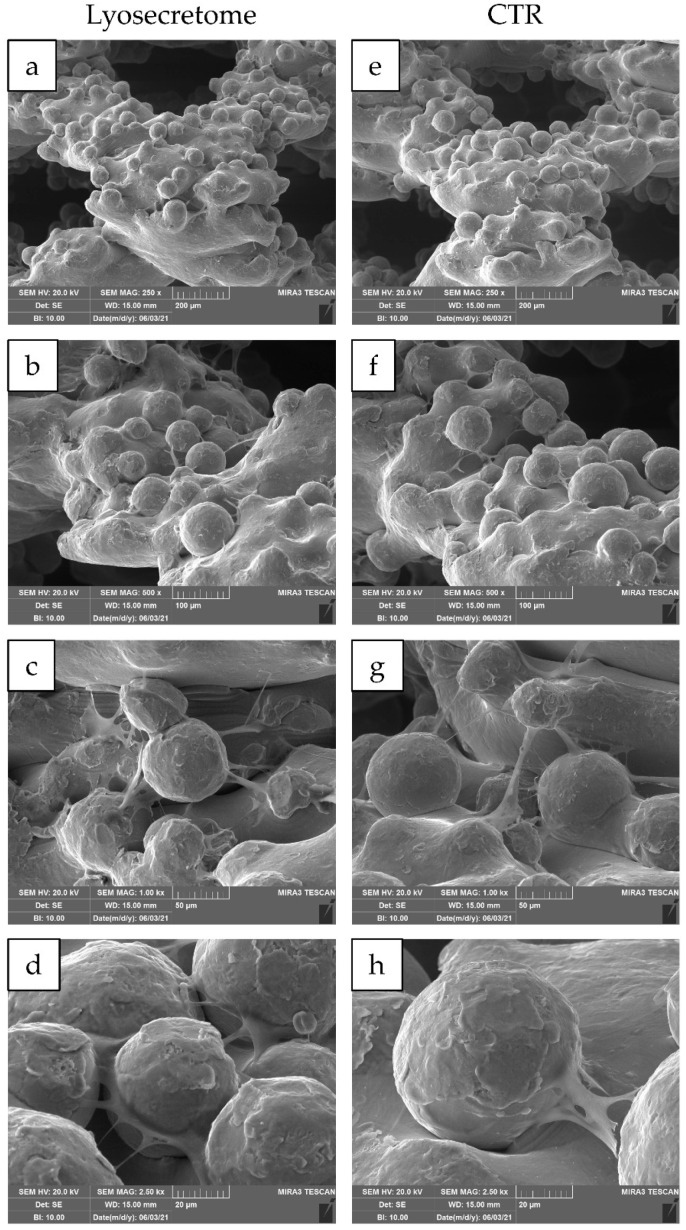
SEM morphological and structural characterizations of titanium cages seeded with MSCs and cultured in osteogenic medium with Lyosecretome or without (CTR) for 7 days. Magnifications: 250× (**a**,**e**), 500× (**b**,**f**), 1000× (**c**,**g**), and 2500× (**d**,**h**). Scale bars: 200 (**a**,**e**), 100 (**b**,**f**), 50 (**c**,**g**), and 20 (**d**,**h**) µm.

**Figure 5:**
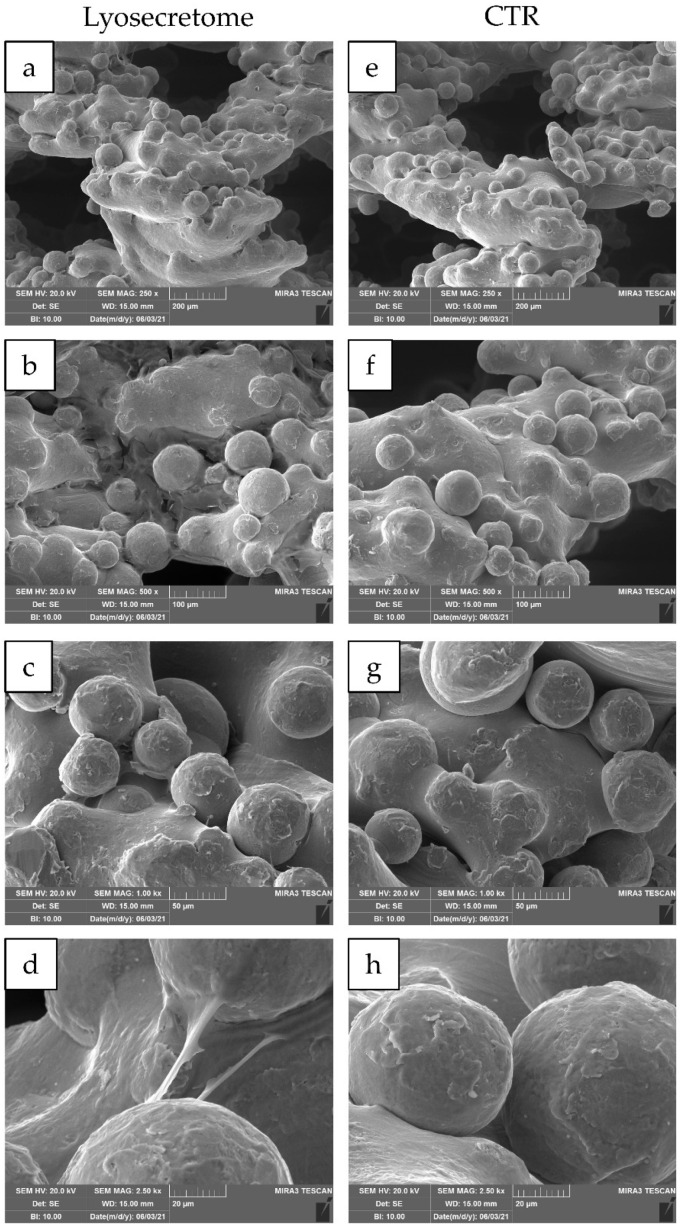
SEM morphological and structural characterizations of titanium cages seeded with MSCs and cultured in osteogenic medium with Lyosecretome or without (CTR) for 14 days. Magnifications: 250× (**a**,**e**), 500× (**b**,**f**), 1000× (**c**,**g**) and 2500× (**d**,**h**). Scale bars: 200 (**a**,**e**), 100 (**b**,**f**), 50 (**c**,**g**), and 20 (**d**,**h**) µm.

**Figure 6:**
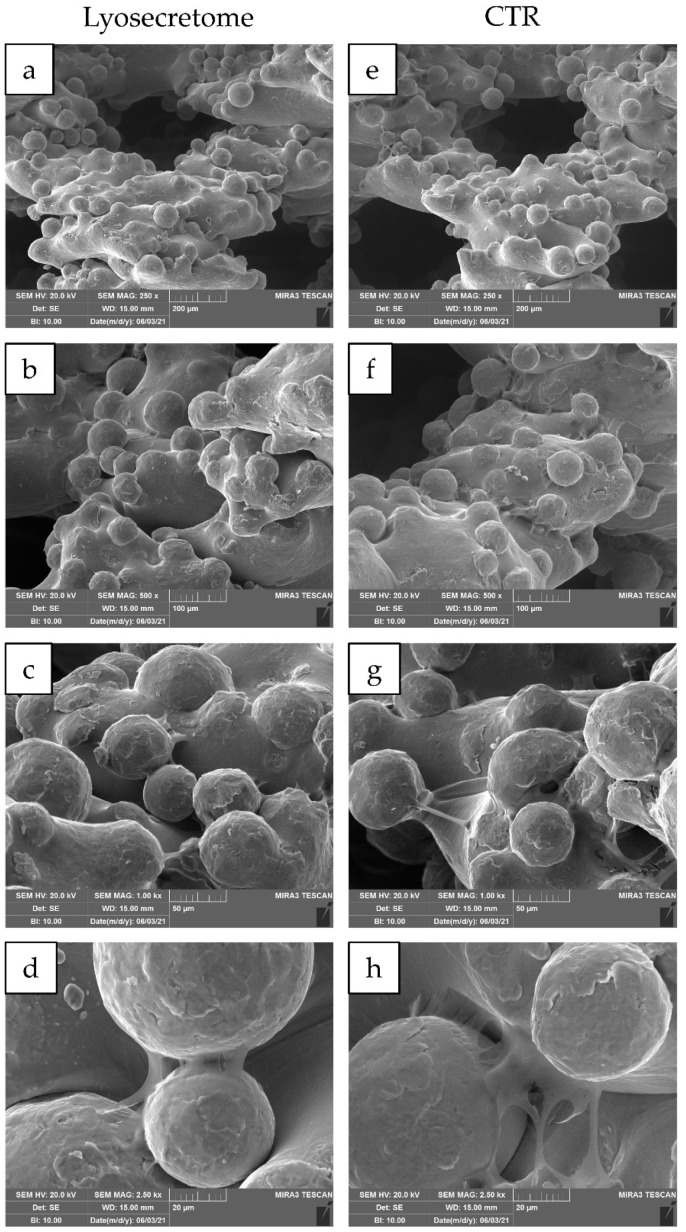
SEM morphological and structural characterizations of titanium cages seeded with MSCs and cultured in osteogenic medium with Lyosecretome or without (CTR) for 21 days. Magnifications: 250× (**a**,**e**), 500× (**b**,**f**), 1000× (**c**,**g**), and 2500× (**d**,**h**). Scale bars: 200 (**a**,**e**), 100 (**b**,**f**), 50 (**c**,**g**), and 20 (**d**,**h**) µm.

24 h after seeding, the cytocompatibility of titanium cages was demonstrated. Indeed, MSCs showed a spread morphology with the initial formation of filopodia (Figure 3). These results are in agreement with the observation conducted by Ragni and colleagues when MSCs were seeded on titanium cages in osteogenic media [26]. After being treated with Lyosecretome, cells showed more frequent and complex cellular processes by forming bridges across the porous surface of the scaffold (Figure 3d). After 7 days, MSCs thoroughly colonized the cages, and the cellular processes became noticeable, even for samples not treated with Lyosecretome (Figure 4). Finally, after 14 (Figure 5) and 21 days (Figure 6), an abundant extracellular matrix was detectable, with a homogeneous and well-organized appearance. Despite this, the porous structure of the scaffold was still distinguishable. No visual differences were revealed after treatment with Lyosecretome; therefore, the treatment with MSC-secretome appears to increase the cage’s colonization by cells in the short time, and SEM detected no apparent differences after 14 or 21 days. Similarly, in previous work, we demonstrated that the presence of Lyosecretome stimulated the colonization of bovine-bone matrix scaffold with a more marked neo-tissue formation after 60 days [19].

Using EDS, a qualitative microanalysis was performed to identify the elements present in the samples from their characteristic X-ray peaks. At 7 days, the following elements were revealed for both conditions (Lyosecretome or CTR): Ti, V, and Al, which are the components of the cage. In addition, the presence of organic material was also revealed by the trace detection of C and O, Ca and P (indicative of the formation of Ca_3_(PO_4_)_2_) on both Lyosecretome and CTR samples at 14 or 21 days (Figure S1). These results agree with the time typically required for the bone matrix to fill with calcium phosphate nanocrystals [39].

**Figure 7:**
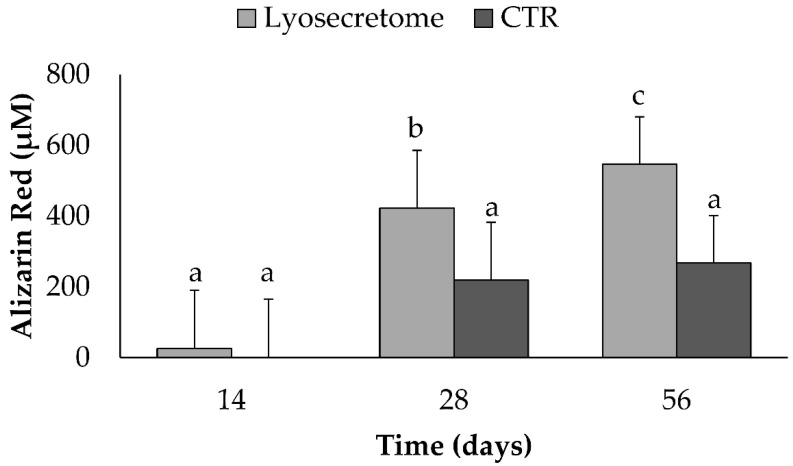
Alizarin red staining for samples cultured in osteogenic differentiation medium with Lyosecretome or without (CTR) for 14, 28, and 56 days. Multifactor ANOVA (time and treatment, mean values ± LSD, *n* = 3) was used. Both time and treatment were significant (*p* < 0.0001 and *p* = 0.0142, respectively). Different letters indicate a significant difference between the means (*p* < 0.05), while the same letters indicate no significant difference between the means (*p* > 0.05).

Alizarin Red staining was performed to demonstrate calcium phosphate deposition by MSCs upon culturing in osteogenic differentiation medium with Lyosecretome or without (CTR) (Figure 7). In Lyosecretome-treated samples, the red color due to alizarin red staining became darker due to increased calcium phosphate deposition, indicating differentiation of MSCs. In detail, after 28 days, a significantly higher amount of alizarin red was detected in the Lyosecretome-treated samples, with respect to 14 days at a time-point (*p* < 0.05). Likely, after 56 days, the mineralization was more significantly increased (*p* < 0.05), also compared with the CTR. The CTR samples showed no significant increase in mineralized matrix deposition at 28 and 56 days (*p* > 0.05).

**Figure 8:**
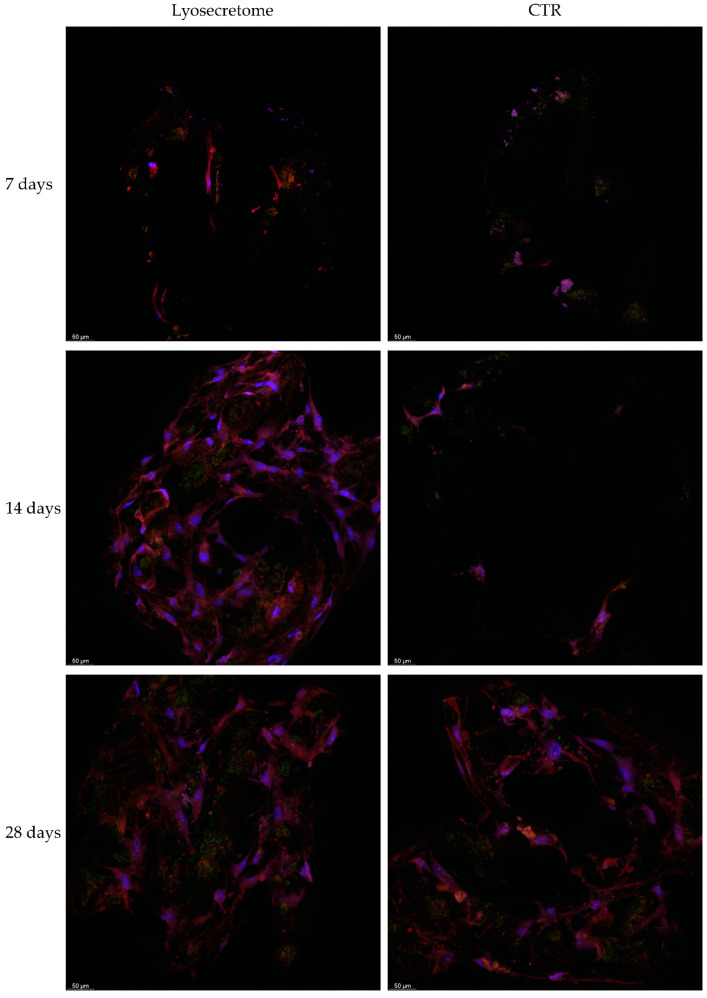
Confocal microscopy images of titanium cages seeded with MSCs and cultured in osteogenic medium with Lyosecretome or without (CTR) for 7, 14, and 28 days. Cell nuclei are stained in blue; actin cytoskeleton is stained in red, and mineralized matrix is stained in green. Scale bar: 50 µm.

**Figure 9:**
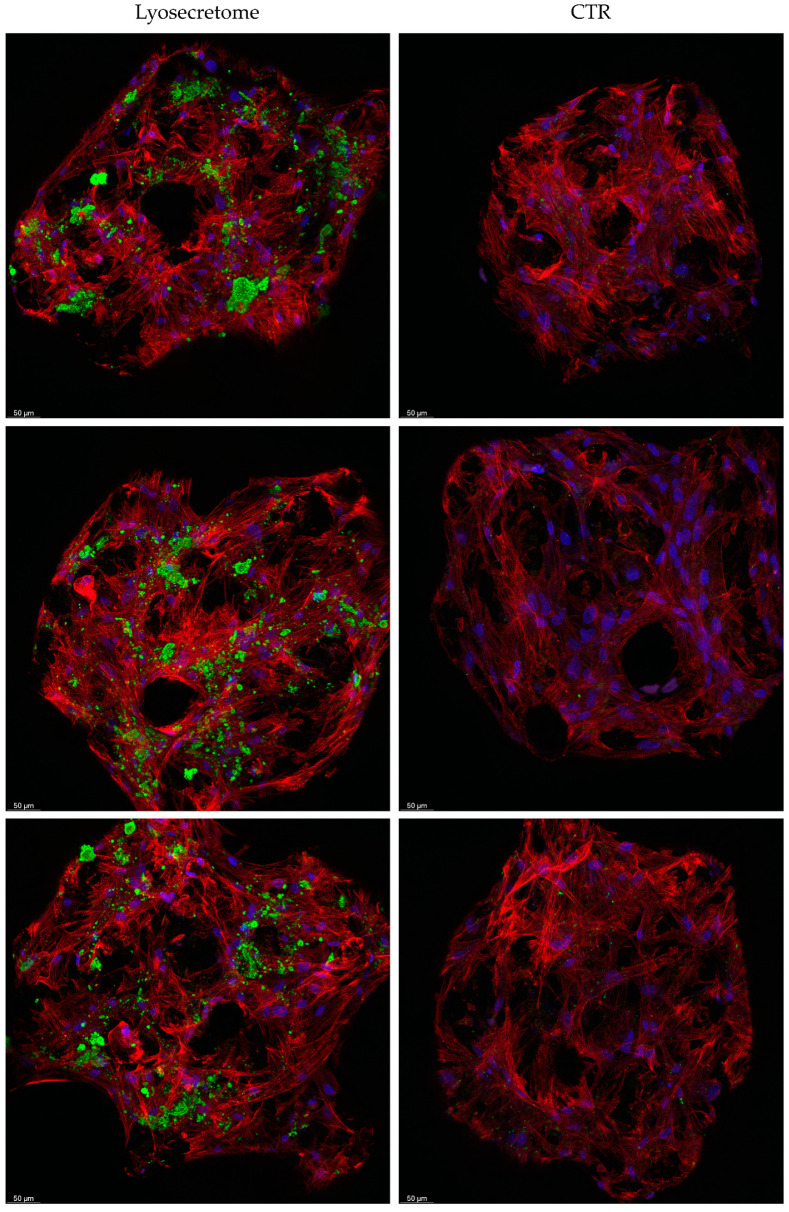
Confocal microscopy images of titanium cages seeded with MSCs and cultured in osteogenic medium with Lyosecretome or without (CTR) for 56 days. Cell nuclei are stained in blue; actin cytoskeleton is stained in red, and mineralized matrix is stained in green. Images are from three different biological replicates. Scale bar: 50 µm.

Analyses with confocal microscopy then confirmed what was observed with SEM and alizarin red analyses. Figure 8 reports a comparison of the samples treated or not with Lyosecretome. After 7 days, few cells are detected in the samples, indicating partial colonization of the scaffolds according to SEM images. Starting from 14 days, the sample treated with Lyosecretome showed more marked scaffold colonization, which is likely a consequence of the demonstrated proliferative effect of MSC-secretome. After 56 days, the morphology of cells was changed, suggesting that MSCs have completed their differentiation towards osteoblasts (Figure 9). Moreover, the presence of mineralized matrix was revealed by Osteoimage^TM^ coloration after 56 days but only in the presence of Lyosecretome (despite the same number of cells and amount of actin). This result confirms the significant difference observed in the alizarin red assay between Lyosecretome and CTR samples at 56 days. It is worth noting that the differentiation of MSCs in osteoblasts induced by Lyosecretome, and the consequent Ca_3_(PO_4_)_2_ deposition, were detected on the entire surface of the scaffold. Conversely, for CTR samples, the mineralized matrix was hard to detect over the whole scaffold, as observed by the images reported in Figure 9. Using 3D reconstruction (Figure S2), it was noted that cells mainly colonize the pore of the scaffolds, where the more mineralized matrix was also detected. In this regard, previous in vivo results showed that an increase of porosity and pore size positively influenced the osteoconductive properties of the scaffold, likely as a consequence of the increased permeability of nutrients and differentiating factors [38].

**Figure 10:**
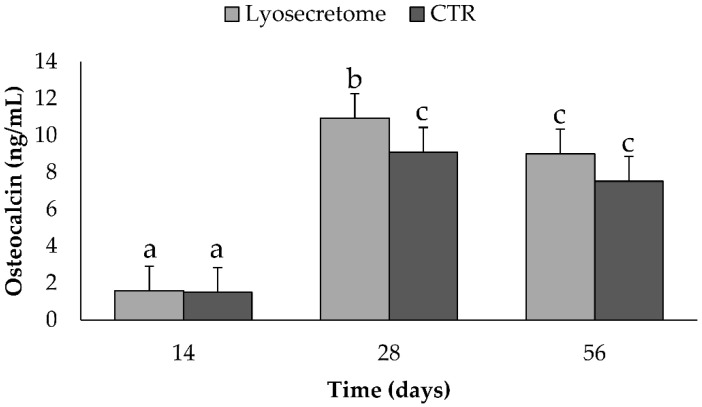
Osteocalcin dosages for samples cultured in osteogenic differentiation medium with Lyosecretome or without (CTR) for 14, 28, and 56 days. Multifactor ANOVA (time and treatment, mean values ± LSD, *n* = 3) was used. Both time and treatment were significant (*p* < 0.0001 and *p* = 0.0440, respectively). Different letters indicate a significant difference between the means (*p* < 0.05), while the same letters indicate no significant difference between the means (*p* > 0.05).

Following osteogenic differentiation, MSCs express specific bone matrix proteins secreted by osteoblasts, such as osteocalcin (OCN), which was dosed on cell culture supernatants (Figure 10). The production of OCN increased significantly after a culture period of 28 days (*p* < 0.05) in osteogenic differentiation medium with Lyosecretome or without (CTR). Also, at 28 days, a significantly higher amount of OCN was dosed in samples treated with Lyosecretome (*p* < 0.05). The higher expression of OCN is in accordance with the higher deposition of mineralized matrix observed by alizarin red for Lyosecretome samples after 28 days. OCN is a bone-specific protein and regulates the mineralization of cells and the formation of mineralized nodules [40,41,42]. Therefore, osteoblasts produce OCN before the mineralized matrix is deposited. In light of this, it is not surprising that the levels of OCN did not increase from 28 to 56 days whereas the mineralized matrix increased, as revealed by confocal microscopy.

These results collectively confirm the role of MSC-secretome in inducing bone regeneration, according to previously published research studies [43,44,45,46,47,48]. This effect mainly depends on the osteogenic factors revealed in Lyosecretome by proteomic characterization [21], such as fibronectin [49], alpha-2-macroglobulin [50], apolipoprotein A [51], and TGF-β [52]. Of note, the MSC-secretome employed in this work was formulated as a ready-to-use, freeze-dried pharmaceutical dosage form, which can be easily adapted by the clinical community. However, despite the solid proof of concept demonstrated in vitro, further studies are required to investigate the bone fracture healing of titanium cages associated with Lyosecretome in vivo.

## 3. Materials and Methods

### 3.1. Materials

Antibiotics, fetal bovine serum (FBS), and culture media were purchased from Euroclone in Milan, Italy. Additional purchases included 3-(4,5-dimethylthiazole-2-yl)-2,5-diphenyl tetrazolium bromide (MTT), β-glycerophosphate, alizarin red, ascorbic acid, bovine serum albumin (BSA), cetylpyridinium chloride, dexamethasone, ethanol, glutaraldehyde, Hoechst 33258, mannitol, Nile Red, phosphatidylcholine (PC), TRITC-phalloidin, and Triton X-100 from Sigma–Aldrich in Milan, Italy. The Osteoimage^TM^ mineralization assay was purchased from Lonza in Milan, Italy. Unless otherwise specified, all the reagents were of analytical grade. An ELISA kit for the dosage of OCN was bought from RayBiotech Life, Inc. in Peachtree Corners, GA, USA.

### 3.2. Isolation and Expansion of Human AD-MSCs

Adipose tissue was harvested from healthy donors undergoing abdominoplasty after informed consent (ASST Grande Ospedale Metropolitano Niguarda, Milan, Italy, Ref. 12 November 2009) and processed as previously reported [53,54] to collect adipose-derived MSCs (AD-MSCs). MSCs were then seeded into flasks (10,000 cells/cm^2^) at 37 °C and 5% CO_2_ and cultured in complete culture medium (DMEM/F12 minimal medium plus 10% *v*/*v* FBS, plus 1% *v*/*v* penicillin/streptomycin and 1% *v*/*v* amphotericin B) until passage three. All the MSCs used fulfilled the requirements for clinical use in terms of identity (according to the International Society for Cellular Therapy [55]) and sterility (according to Eu. Ph. 9.0, 2.6.27).

### 3.3. Lyosecretome Preparation and Characterization

Lyosecretome was prepared according to previously reported procedures [21,22] in GMP-compliant conditions. Briefly, secretome release was induced by culturing MSCs in DMEM/F12 without FBS for 48 h. Conditioned media were collected, centrifuged at 3500× *g* for 10 min, and then concentrated and purified (diafiltered) by tangential flow filtration (KrosFlo^®^ Research 2i system, Spectrum Laboratories, Milan, Italy). The shear rate of the feed stream was maintained between 2000 s^−1^ and 6000 s^−1^, and trans-membrane pressure did not exceed 10 psi. Both soluble proteins and EVs of MSC-secretome were retained as a 5 kDa Molecular Weight Cut Off (MWCO) filtration module (Spectrum Laboratories, Milan, Italy), with a superficial area of 0.235 cm^2^. Mannitol was dissolved as a cryoprotectant (0.5% *w*/*v*); the resulting solution was frozen at −80 °C and freeze-dried (Christ Epsilon 2-16D LSCplus) at 8 × 10^−1^ mbar and −50 °C for 72 h. Each milligram of the freeze-dried product contained 1 × 10^6^ cell equivalents. Lyosecretome was stored at −20 °C for 5 months until use. Lyosecretome was characterized as follows.

#### 3.3.1. Total Protein and Lipid Content

Total proteins and lipids were quantified by the BCA Protein Assay Kit (Thermo Fisher Scientific, Milan, Italy) and the Nile Red method, respectively, as previously reported and validated [21]. The protein (or lipid) concentration was extrapolated from a concentration vs. absorbance plot obtained from standard BSA (or PC) solutions, using a third-degree polynomial equation, where R^2^ = 0.99.

#### 3.3.2. EV Particle Size and Concentration

The particle size and concentration of EVs were determined by Nanoparticle Tracking Analysis (NTA, NanoSight NS 300 equipment, Malvern Panalytical Ltd., Malvern, UK). Lyosecretome was dispersed in water at 1 mg/mL and analyzed at room temperature with a detection angle of 90°. Measurements were conducted in triplicate, and the software NTA elaborated on the data.

#### 3.3.3. Microbiological Controls

Lyosecretome was tested for sterility, endotoxins, mycoplasma, and microbiological contaminations, as described in the current version of European Pharmacopoeia. In detail, sterility and microbiological tests were performed as indicated in the EuPh 2.6.27 and 2.6.1 chapters, respectively. Bacterial endotoxins evaluation was instead conducted by the Limulus amebocyte lysate test (EuPh 2.6.14) and measured by endotoxin unit (EU). Mycoplasma contamination was investigated by performing the NAT test (EuPh 2.6.7).

### 3.4. Titanium Cages

Titanium cages were kindly provided by the company MT Ortho (MT Ortho s.r.l., Aci Sant’Antonio, Catania, Italy). Cages were manufactured by an additive manufacturing technology called electron beam melting, where an electron beam is used to melt titanium powder that is then deposited layer-by-layer onto the base plate. Scaffolds were 1 × 1 × 0.3 cm in size. Each cage was sterilized by autoclaving (Systec V-65, Wittenberg, Germany) at 121 °C and 2 atm for 20 min.

### 3.5. Seeding of AD-MSCs

90,000 MSCs were seeded onto the upper surface of each cage placed inside a 24 well-plate. The porous substrates absorbed the cellular suspension in a humidified atmosphere of 95% air with 5% CO_2_ at 37 °C for 2 h. Afterward, complete culture media was added to each well. 24 h after seeding, scaffolds were moved from the original wells to new wells to avoid residual cells on the plastic surface below. During all the experiments, the cell/cage constructs were incubated in a humidified atmosphere of 95% air with 5% CO_2_ at 37 °C.

### 3.6. Evaluation of Cell Proliferation

Lyosecretome was previously solubilized in culture media without FBS and then added to each well’s cell/cage constructs. The following concentrations were considered: 0, 200,000 and 400,000 cell equivalents per well. Each condition was tested in triplicate. In order to evaluate the mitochondrial activity during the culture period, a test with MTT was performed on days 5, 9, and 14, as previously reported [56]. The cell metabolic activity was calculated according to the following equation:

Metabolic activity % = 100 × (Abs_sample_/Abs_control_) (1)

where Abs_sample_ is the mean value of the measured absorbance of the tested samples, and Abs_control_ is the mean value of the measured absorbance of cells not incubated with Lyosecretome.

### 3.7. MSCs Culture and Osteogenic Differentiation

In order to induce osteogenic differentiation, the cell/cage constructs were moved into osteogenic medium, prepared with 2% *v*/*v* FBS, dexamethasone (5 nM), ascorbic acid (2.5 μg/mL), and β-glycerophosphate (0.5 mM). Lyosecretome was added to half of the samples at the final concentration of 2 × 10^6^ cell equivalents per well. The other samples were cultured with the only osteogenic medium (CTR).

### 3.8. Scanning Electron Microscopy (SEM)

After 24 h and 7, 14, and 21 days of osteogenic differentiation, with or without Lyosecretome, the cell/cage constructs were washed with PBS and fixed for 3 h with 3% *v*/*v* glutaraldehyde at 4 °C. Afterward, the scaffolds were dehydrated with graded ethanol series, starting with 50, 70, 90, and 100% *v*/*v*. The samples were then attached to stubs by a conductive adhesive carbon tape and metal-coated with 10 nm chromium using a high-vacuum Quorum Q150T ES Plus sputtering system. SEM imaging was performed with SEM MIRA3 (Tescan, Brno, Czech Republic) operating with an acceleration voltage of 5 kV and an EDS detector (X-max 50 mm^2^, Oxford Instruments, Oxford, UK). Each condition was tested in three independent experiments.

### 3.9. Alizarin Red Assay

After 14, 28, and 56 days of osteogenic differentiation, with or without Lyosecretome, the cell/cage constructs were stained with alizarin red to reveal the deposition of calcium-rich mineralized matrix. Briefly, each sample was washed with PBS, fixed with 70% *v*/*v* ethanol for 60 min at room temperature, and stained for 10 min with alizarin red 40 mM at pH 4–4.2. Samples were then rinsed with distilled water five times and once with PBS for 10 min. Finally, samples were treated with 10% *w*/*v* cetylpyridinium chloride for 15 min at room temperature; the alizarin red concentration was extrapolated from a concentration vs. absorbance plot obtained from standard alizarin red solutions, using a third-degree polynomial equation, where R^2^ = 0.99. Each condition was tested in three independent experiments.

### 3.10. Confocal Microscopy

After 7, 14, 28, and 56 days of osteogenic differentiation, with or without Lyosecretome, the cell/cage constructs were washed with PBS and fixed for 3 h with 3% *v*/*v* glutaraldehyde at 4 °C. Actin cytoskeleton was stained with TRITC-phalloidin diluted 1:7000 in PBS containing 0.1% *v*/*v* Triton X-100, 0.1% *w*/*v* BSA and 10% *v*/*v* FBS. Cell nuclei were stained with 100 µL of Hoechst 33258, diluted 1:10,000 in PBS. Hydroxyapatite was stained with Osteoimage^TM^ mineralization assay according to the manufacturer’s instruction. Scaffolds were placed on a microscope slide and imaged using a Confocal Laser Scanning Microscope (CLSM) (Leica TCS SP2, Leica Microsystems, Wetzlar, Germany) with λ_ex_ = 540/5 nm and λ_em_ = 570/3 nm for TRITC-phalloidin, λ_ex_ = 346 nm and λ_em_ = 460 nm for Hoechst 33258 and λ_ex_ = 492 nm and λ_em_ = 520 nm for Osteoimage^TM^. The acquired images were processed using the software associated with the microscope (Leica Microsystem, Wetzlar, Germany). Each condition was tested in three independent experiments.

### 3.11. Dosage of Osteocalcin (OCN) by ELISA

At 14, 28, and 56 days, OCN was dosed in cell supernatants by an ELISA kit according to the manufacturer instructions. A calibration curve was built using OCN standards in the range of 0–100 ng/mL. Each condition was tested in three independent experiments.

### 3.12. Statistical Analysis

Raw data were processed through STATGRAPHICS XVII (Statpoint Techonologies, Inc., Warrenton, VA, USA). A general linear analysis of variance model (ANOVA) was coupled with an LSD (Least Significant Difference) test to estimate the differences between means. Proliferation data were elaborated in detail, considering the Lyosecretome concentration and time as fixed factors with cell metabolic activity (%) as the response variable. In addition, data regarding the alizarin red assay were elaborated considering the time and Lyosecretome treatment as fixed factors with the alizarin red concentration as the response variable. Finally, the OCN data were elaborated considering the time and Lyosecretome treatment as fixed factors with the ng of OCN as the response variable. Statistical significance was determined at *p* < 0.05. Unless otherwise specified, data are reported as mean values ± standard deviation (from at least 3 independent experiments).

## Data Availability

The data presented in this study are contained within the article and its supplementary materials.

## Supplementary Materials

The following are available online at https://www.mdpi.com/article/10.3390/ijms22168445/s1.
