# Supplementary material for: Freeze-Dried Secretome (Lyosecretome) from Mesenchymal Stem/Stromal Cells Promotes the Osteoinductive and Osteoconductive Properties of Titanium Cages
Source: Int J Mol Sci. 2021 Aug 6;22(16):8445. doi: 10.3390/ijms22168445 (PMC8395097; doi:10.3390/ijms22168445)
Supplement: Supplementary file 1 [file ijms-22-08445-s001.zip › ijms-1316758-supplementary.pdf]

Freeze-dried Secretome (Lyosecretome) from Mesenchymal Stem/stromal Cells Promotes the Osteoinductive and Osteoconductive Properties of Titanium Cages

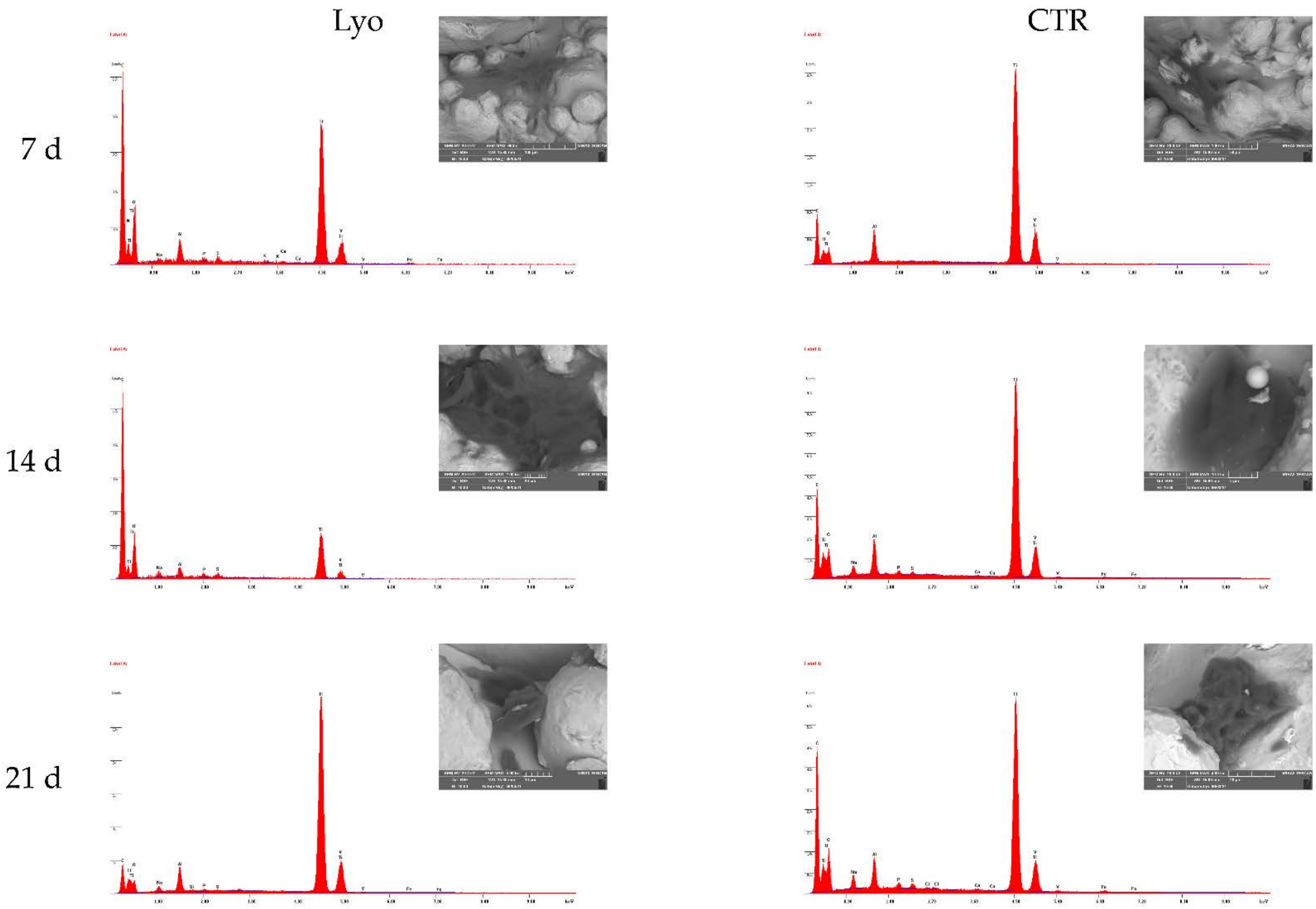

Figure S1. Qualitative microanalysis of the samples conducted by SEM EDS of titanium cages seeded with MSCs, and cultured in osteogenic medium with Lyosecretome or without (CTR) for 7, 14 and 21 days.

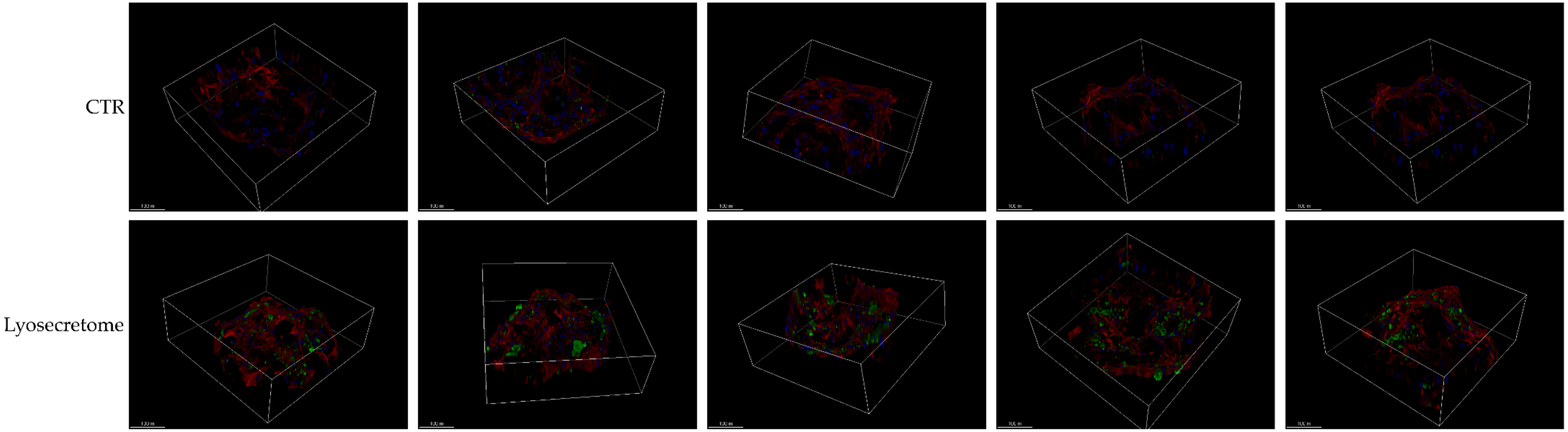

Figure S2. 3D reconstruction by confocal microscopy of the titanium cage pores colonized by MSCs after 56 days of culture in osteogenic medium with Lyosecretome or without (CTR). Cell nuclei are stained in blue, actin cytoskeleton is stained in red and mineralized matrix is stained in green. Scale bar: 100 μm.
